# Universal entanglement growth along imaginary time in quantum critical systems

**DOI:** 10.1038/s41467-026-75645-x

**Published:** 2026-08-03

**Authors:** Chang-Yu Shen, Shuai Yin, Zi-Xiang Li

**Affiliations:** 1 Beijing National Laboratory for Condensed Matter Physics & Institute of Physics, Chinese Academy of Sciences, Beijing, China; 2 University of Chinese Academy of Sciences, Beijing, China; 3 Guangdong Provincial Key Laboratory of Magnetoelectric Physics and Devices, School of Physics, Sun Yat-sen University, Guangzhou, China; 4 School of Physics, Sun Yat-sen University, Guangzhou, China

**Keywords:** Phase transitions and critical phenomena, Statistical physics, Quantum information

## Abstract

Characterizing universal entanglement features in higher-dimensional quantum matter is a central challenge in quantum information science and condensed matter physics. While subleading corner terms in two-dimensional systems encode the essential fingerprints of the underlying conformal field theory, accessing these features remains notoriously difficult compared to the well-understood one-dimensional case. Here, we uncover a universal non-equilibrium scaling law governing imaginary-time entanglement dynamics. We demonstrate that for 2D quantum critical points, the corner entanglement grows logarithmically with imaginary time, with a coefficient dictated solely by the universality class. By validating this framework through large-scale Quantum Monte Carlo simulations, we definitively resolve the entanglement structure of the interacting Gross-Neveu-Yukawa critical point, revealing significant interaction-driven deviations from free-fermion theories. Crucially, our approach extracts high-precision universal data from the early stages of relaxation, thereby bypassing the prohibitive computational bottlenecks of full equilibrium convergence. This work establishes a direct bridge between non-equilibrium critical phenomena and entanglement spectroscopy, providing a robust theoretical blueprint for applications on both classical numerical computation and emerging quantum hardware.

## Abstract (web summary)

Extracting universal entanglement features in higher-dimensional quantum critical systems has remained challenging. Here, the authors demonstrate a universal logarithmic scaling of corner entanglement in imaginary time, enabling efficient determination of critical properties from short-time dynamics.

## Introduction

Unveiling the universal structure of quantum entanglement in strongly correlated matter remains one of the most profound challenges at the interface of condensed matter physics and quantum information science. While the “area law" governs the dominant entanglement scaling in ground states^1^, it is the subleading corrections that encode the quintessential fingerprints of quantum phases and phase transitions. In one-dimensional (1D) critical systems, conformal field theory (CFT) successfully links these logarithmic corrections to the central charge^2–6^. The features of entanglement entropy are more complex and intriguing in higher dimensions. In two-dimensional (2D) topologically ordered phases, a subleading constant term-the topological entanglement entropy-directly reveals the nature of the topological order^7–12^. Similarly, 2D quantum critical points (QCPs) display universal logarithmic corrections, notably arising from sharp corners in the entanglement region, which are tied to the underlying CFT^4,11,13–21^. However, in stark contrast to the well-understood 1D case, the rich entanglement structure of higher-dimensional quantum many-body systems remains far less explored and warrants significant investigation. The corner contribution, for instance, is notoriously difficult to isolate from the overwhelming non-universal background.

Conventionally, probing these universal features relies on calculating the equilibrium ground state, a task that demands converging to the thermodynamic limit. For computational methods like Quantum Monte Carlo (QMC)^22,23^, this poses a “Holy Grail" challenge: extracting a subtle logarithmic signal from noisy data requires astronomical precision, often hindered by the exponential degradation of signal-to-noise ratios^24,25^. Despite significant methodological advances^26–37^, including the development of incremental algorithms^35^, the accurate and efficient determination of the universal coefficient for the corner term, especially in interacting fermionic systems, persists as a major challenge. More fundamentally, this static perspective overlooks a vast and fertile territory: non-equilibrium dynamics. With the rapid advent of quantum simulators, imaginary-time evolution has evolved from a cornerstone of computational many-body physics into a distinct experimental reality for ground-state preparation on quantum hardware^38–41^. Although recent research has made progress in illuminating non-equilibrium criticality^42–50^, a theoretical framework governing entanglement growth during imaginary-time evolution in 2D systems is entirely missing. Establishing such a theory is imperative: it provides the necessary framework to guide and benchmark quantum simulation experiments, while simultaneously offering a powerful route to bypass the computational bottlenecks of equilibrium calculations.

**Fig. 1:**
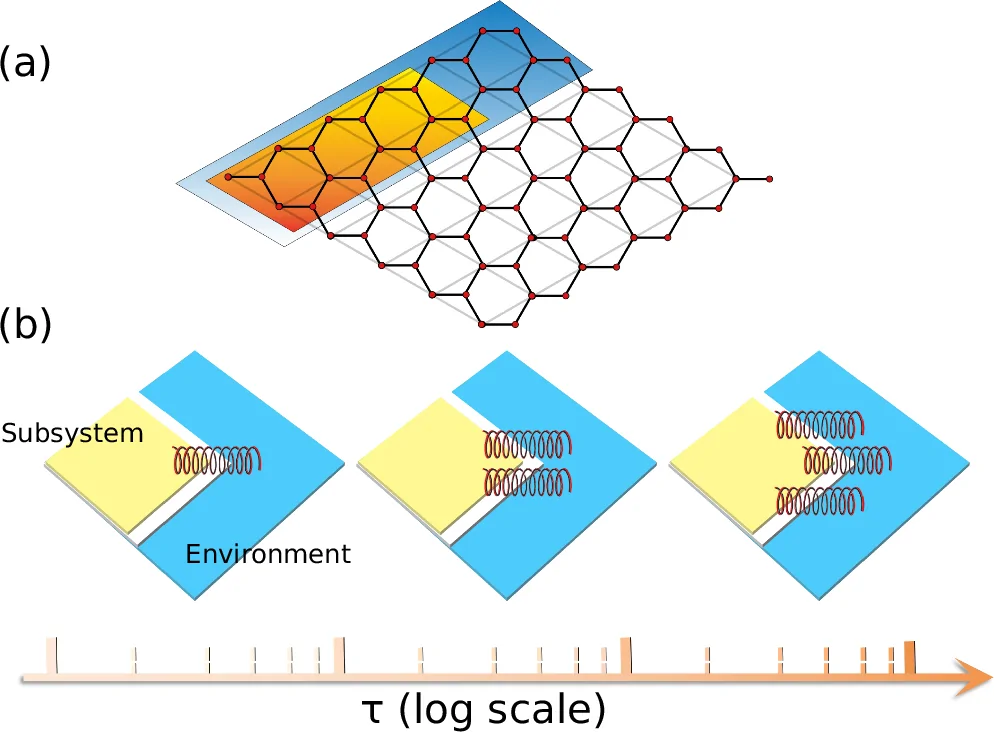
Subsystem geometries and corner entanglement dynamics. **a** Schematic of the lattices and subsystem cuts used for entanglement entropy (EE) calculations in this Letter. The full system has periodic boundary conditions in both horizontal and vertical directions. The orange region (Region *A*) has dimensions $\frac{L}{3}\times \frac{2L}{3}$ and features two $\frac{\pi }{3}$ and two $\frac{2\pi }{3}$ corners. The blue region (Region *B*) has dimensions $\frac{L}{3}\times L$ and possesses a smooth boundary. Crucially, both regions share the same boundary length. **b** Schematic illustration of the corner entanglement growth in imaginary-time evolution. The springs represent the degree of corner contribution to entanglement between the subsystem and the environment. In the non-equilibrium regime, the corner contribution to the entanglement entropy increases linearly with the logarithm of the imaginary time *τ*.

Here, we bridge these frontiers by investigating the imaginary-time entanglement dynamics of quantum critical systems. We identify a universal non-equilibrium scaling law that governs the growth of corner entanglement entropy. Unlike previous studies strictly confined to 1D, we demonstrate that in 2D QCPs, the corner entanglement grows logarithmically with imaginary time, with a coefficient determined solely by the universality class of the underlying CFT, as schematically illustrated in Fig. 1. This discovery constitutes a paradigm shift: it establishes that static universal entanglement features- traditionally believed to be accessible only in the equilibrium ground state-can be rigorously extracted from the short-time non-equilibrium dynamics.

We validate this theoretical framework through unbiased large-scale QMC simulations. By bypassing the need for full equilibrium convergence, our approach reduces the computational cost by orders of magnitude. We demonstrate the power of this protocol by resolving the entanglement structure of the interacting Gross-Neveu-Yukawa (GNY) critical point^51–59^, providing a high-precision benchmark that highlights significant deviations from free-fermion theories. To conclude, our work establishes a robust framework for probing universal entanglement properties in the short-time regime. This not only advances the fundamental theory of non-equilibrium criticality but also but also provide a highly efficient numerical protocol for entanglement spectroscopy. Furthermore, our theoretical discovery can be directly implemented and verified on existing quantum hardware, bridging the gap between abstract scaling laws and state-of-the-art quantum simulation.

## Result

### Universal non-equilibrium scaling of entanglement entropy

In 2D quantum critical systems, the entanglement entropy encodes the universal properties of the critical point. Specifically, for critical systems without a Fermi surface, the second Rényi entropy for a subregion *A* with linear size *L* follows the scaling form^14,16,19,60^:

1 $${S}_{2}^{A}(L)=aL-{s}_{c}(\theta ) \; {{{\rm{ln}}}} \; L+\,{\mbox{const}}\,.+{{{\mathcal{O}}}}(1/L).$$

Here, *aL* represents the standard area-law contribution found in generic quantum ground states, where the coefficient *a* is non-universal and depends on microscopic details. The universal component is the logarithmic correction arising from the sharp corner in region *A*. The angle-dependent coefficient *s*_*c*_(*θ*) is independent of microscopic specifics and is determined solely by the universality class of the quantum critical systems.

Here, we investigate the scaling behavior of the entanglement entropy during imaginary-time evolution from a product initial state. In the relaxation process, the wave function $\left.| \psi (\tau )\right\rangle$ evolves according to the imaginary-time Schrödinger equation $-\frac{\partial }{\partial \tau }\left.| \psi (\tau )\right\rangle=H\left.| \psi (\tau )\right\rangle$, subject to wavefunction normalization. We focus on the universal component of the entanglement entropy—specifically the corner correction encoding critical properties—which we denote as $\Delta {S}_{2}^{A}$. Motivated by the equilibrium scaling form in Eq. (1), we propose a general ansatz for the corner contribution $\Delta {S}_{2}^{A}$ during imaginary-time evolution:

2 $$\Delta {S}_{2}^{A}(L,\tau )={s}_{c}(\theta ) \; {{{\rm{ln}}}} \; L+{{{\mathcal{F}}}}(\tau /{L}^{z})+\,{\mbox{const}}\,.+{{{\mathcal{O}}}}(1/L),$$

where *z* is the dynamic exponent of the quantum critical point (QCP) and ${{{\mathcal{F}}}}$ is a universal scaling function. The system relaxes to the ground state in the limit *τ* ≫ *L*^*z*^, for which the scaling behavior must recover Eq. (1).

Conversely, in the short-time regime where *τ* ≪ *L*^*z*^, we speculate that starting from a product state, the spread of entanglement is confined by a finite length scale *ξ* ~ *τ*^1/*z*^ and has not yet reached the system boundary. Consequently, $\Delta {S}_{2}^{A}(L,\tau )$ must scale with the correlation length *ξ* rather than the subregion size *L*. This implies that the dependence on *L* in Eq. (2) must vanish in the short-time regime. This physical requirement dictates the asymptotic behavior of the scaling function: ${{{\mathcal{F}}}}(x)\to \frac{{s}_{c}(\theta )}{z}{{{\rm{ln}}}}(x)$ as *x* → 0. Therefore, in the early stages of evolution, we expect the universal corner contribution to obey the following scaling form:

3 $$\Delta {S}_{2}^{A}(L,\tau )=\frac{{s}_{c}(\theta )}{z} \; {{{\rm{ln}}}} \; \tau+\,{\mbox{const}}\,.+{{{\mathcal{O}}}}(1/L).$$

Notably, in this regime, $\Delta {S}_{2}^{A}(L,\tau )$ is independent of the system size to leading order, as the correlation length remains much smaller than *L*. Hence, this short-time scaling form offers a powerful tool to extract the universal coefficient *s*_*c*_(*θ*), which characterizes the critical properties of the QCP.

## Model

We select a classic model of interacting fermions in two dimensions as the benchmark system for our new method: the Hubbard model on a honeycomb lattice at half filling. Its Hamiltonian is given by:

4 $$H=	 {H}_{t}+{H}_{u}\\=	 -t {\sum}_{\langle ij \rangle,\sigma }{c}_{i \sigma }^{{{\dagger}} }{c}_{j \sigma }+\frac{U}{2}{\sum}_{i}{({n}_{i\uparrow }+{n}_{i\downarrow }-1)}^{2},$$

in which *t* is the hopping coefficient and *U* represents the strength of the Hubbard interaction. The restriction to half filling ensures the absence of the notorious “sign problem” in QMC simulations^61–68^. This model is known to host a quantum phase transition from a Dirac semi-metal to an AFM insulator at a critical interaction strength *U*_*c*_ ≈ 3.8 ^53,69,70^. In the AFM phase, the system undergoes spontaneous symmetry breaking from SU(2) down to U(1), resulting in *N*_*G*_ = 2 Goldstone modes. Crucially, the effective field theory of model (4) possesses a Lorentz-invariant structure with a dynamic critical exponent *z* = 1. This feature implies that the critical dynamics are governed by a relativistic CFT, where time and space scale identically (*τ* ~ *L*).

### Free Dirac-fermion systems

Before analyzing the interacting critical point, we benchmark the non-equilibrium scaling using the exactly solvable non-interacting (*U* = 0) Hubbard model. This system hosts four Dirac cones-two spin and two valley flavors-implying a total theoretical corner-entanglement coefficient *s*_*c*_ = 0.3116, obtained by summing the CFT contributions ${a}_{2}^{{{{\rm{f}}}}}(\pi /3)=0.0330$ and ${a}_{2}^{{{{\rm{f}}}}}(2\pi /3)=0.0059$^21^.

**Fig. 2:**
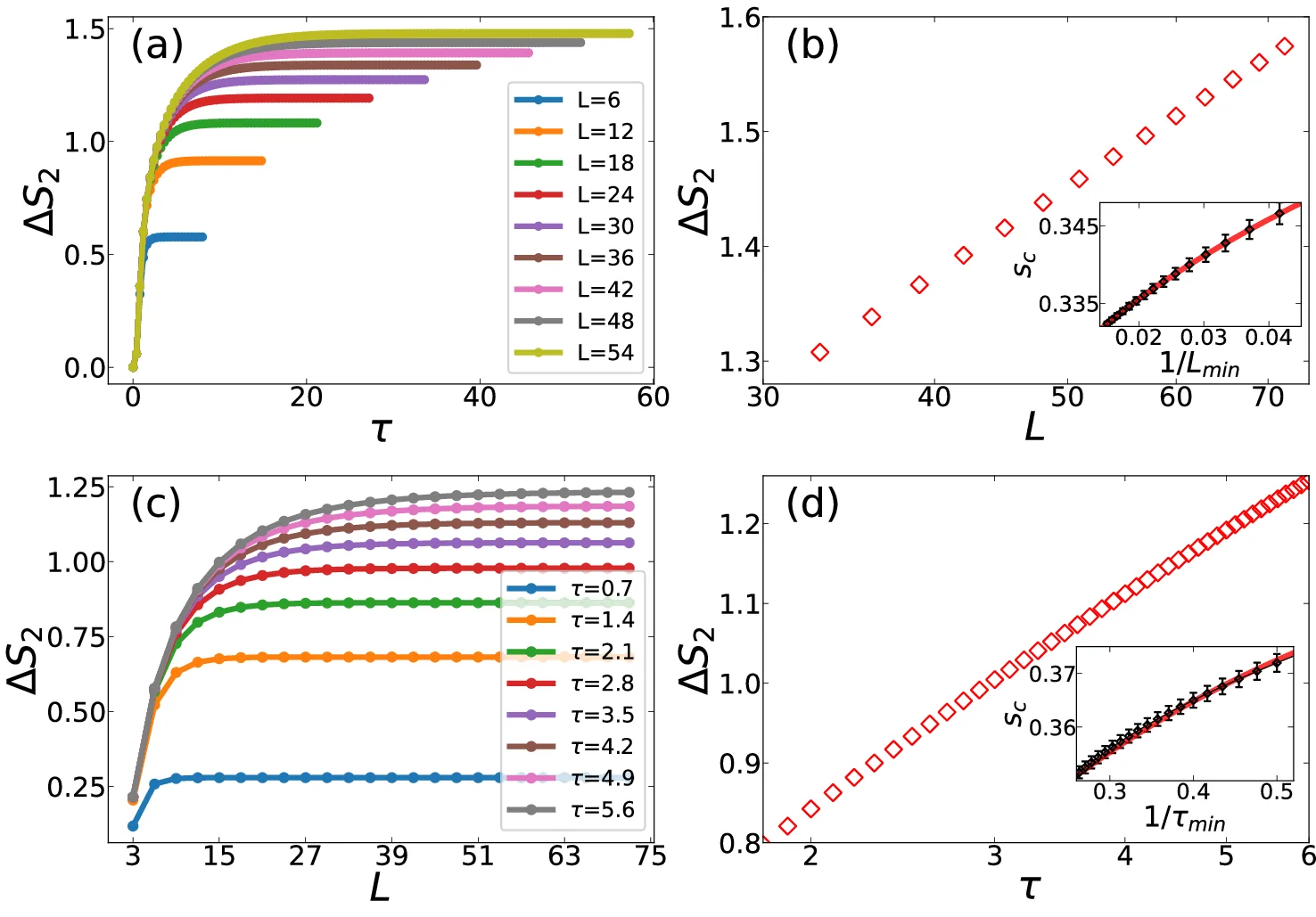
Non-equilibrium relaxation and scaling of the corner entanglement entropy Δ*S*_2_ in the non-interacting (*U* = 0) system. Error bars for all data points in this figure are exactly zero. **a** Imaginary-time evolution of Δ*S*_2_ from a product state toward the free Dirac fermion ground state. **b** Scaling analysis of the equilibrium corner entanglement Δ*S*_2_ with system size *L*, demonstrating the logarithmic dependence of Δ*S*_2_ on *L*. We determine the equilibrium coefficient ${s}_{c}^{{{{\rm{eq}}}}}$ by fitting Δ*S*_2_(*L*) to the form *s*_*c*_ ln *L* + const. over fitting windows with varying lower bounds *L*_min_ and a fixed maximum *L*_max_ = 90. Inset: Extrapolation of the fitting results of ${s}_{c}^{{{{\rm{eq}}}}}({L}_{{{{\rm{min}}}}})$ to ${L}_{\,{\mbox{min}}\,}^{-1}\to 0$ using a polynomial fit, yielding ${s}_{c}^{{{{\rm{eq}}}}}=0.3116(13)$. **c** Δ*S*_2_ plotted against system size *L* for different fixed values of imaginary time *τ*. In the non-equilibrium relaxation regime (*τ* ≪ *L*), Δ*S*_2_ exhibits a “size-independent plateau” determined solely by *τ*. **d** The universal size-independent scaling of Δ*S*_2_ plotted as a function of imaginary time *τ*. Inset: We extract the coefficient by fitting Δ*S*_2_(*τ*) to the logarithmic form *s*_*c*_ln*τ* + const. in fitting windows with varying minima *τ*_min_ and a fixed *τ*_max_ = 6.0. Extrapolating to ${\tau }_{\,{\mbox{min}}\,}^{-1}\to 0$ yields ${s}_{c}^{{{{\rm{neq}}}}}=0.3111(15)$.

We initialize the imaginary-time evolution from an AFM product state, for which Δ*S*_2_ = 0. The subsequent relaxation of Δ*S*_2_ displays two distinct regimes. For fixed system size *L*, the quantity grows rapidly at short imaginary times and eventually saturates for *τ* ≫ *L*, yielding the ground-state value as shown in Fig. 2a. To extract the equilibrium coefficient, we fit the saturated values Δ*S*_2_(*L*) as shown in the inset of Fig. 2b to the logarithmic form Δ*S*_2_ = *s*_*c*_ ln *L* + const. As shown in Fig. 2b, we use fitting windows $[{L}_{\min },{L}_{\max }]$ with a fixed upper bound ${L}_{\max }=90$, and for each choice of ${L}_{\min }$, all system sizes $L\ge {L}_{\min }$ are included in the fit. The resulting size-dependent coefficients converge smoothly as ${L}_{\min }$ increases, producing the extrapolated equilibrium value ${s}_{c}^{{{{\rm{eq}}}}}=0.3116(13)$.

To probe the non-equilibrium scaling, we fix *τ* and study the dependence of Δ*S*_2_ on the system size in Fig. 2c. In the regime *τ* ≪ *L*, the results exhibit a clear size-independent plateau, consistent with the expectation that Δ*S*_2_ depends only on *τ* before the system relaxes to its equilibrium state Eq. (3). By plotting these plateau values in the inset of Fig. 2d, we can see a robust linear dependence for these plateau values versus ln*τ*. The finite-time coefficient is obtained by fitting Δ*S*_2_(*τ*) = *s*_*c*_ln*τ* + const. over fitting windows $[{\tau }_{\min },{\tau }_{\max }]$ with a fixed upper bound ${\tau }_{\max }=6.0$, and extrapolating the extracted coefficients to ${\tau }_{\min }^{-1}\to 0$ as shown in Fig. 2d. This procedure yields ${s}_{c}^{{{{\rm{neq}}}}}=0.3111(15)$, in excellent agreement with both the equilibrium result and the field-theoretic prediction.

Our non-equilibrium result, ${s}_{c}^{{{{\rm{neq}}}}}$, aligns closely with the CFT prediction *s*_*c*_ = 0.3116 for free Dirac fermions^21^. While both approaches suffer from finite-size effects, the non-equilibrium scaling converges significantly faster: stable universal coefficients are obtained with system sizes as small as *L* = 54 for imaginary times *τ*≤6.0. In contrast, the equilibrium analysis requires substantially larger lattices (up to *L* = 90) and corresponding long imaginary time (*τ* ~ *L*) to achieve comparable precision. This demonstrates a practical advantage of the non-equilibrium method for extracting universal entanglement coefficients in 2D fermion systems.

### Gross-Neveu-Yukawa quantum critical point

We now extend this non-equilibrium protocol to the interacting GNY QCP, where the universal coefficient *s*_*c*_ lacks a known analytical solution. While recent QMC studies have investigated *s*_*c*_ at the GNY QCP^35,71^, those works focused on subsystem corners with angles of *π*/3 and *π*/4, respectively. In contrast, our setup utilizes a subsystem geometry on the honeycomb lattice that incorporates both *π*/3 and 2*π*/3 corners, thereby probing a different set of universal corner contributions. Characterizing universal properties in this interacting regime via conventional equilibrium projector QMC is notoriously difficult due to critical slowing down. This typically demands prohibitive computational costs (e.g., *τ* ~ 2*L* for *L* ≥ 18) to reliably converge to the ground state in the thermodynamic limit^35^.

**Fig. 3:**
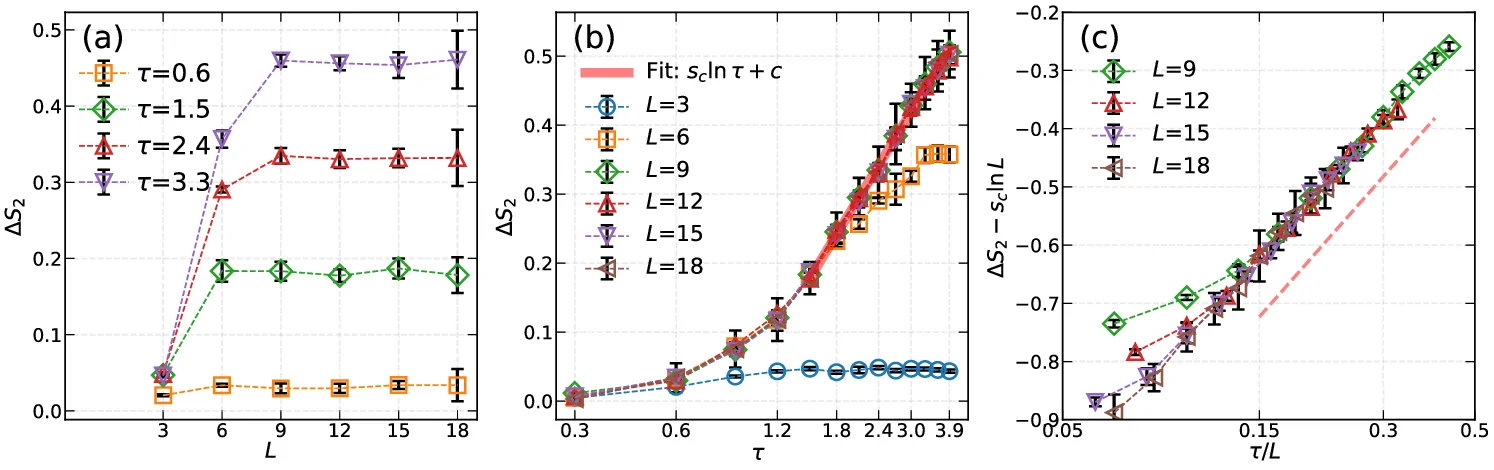
Non-equilibrium dynamics of Δ*S*_2_ at the GNY QCP (*U* = 3.8). All simulations start from an AFM product state. **a** Δ*S*_2_ versus system size *L* for different *τ*. In the non-equilibrium regime (*τ* ≪ *L*), Δ*S*_2_ exhibits a size-independent plateau. **b** Logarithm plot of Δ*S*_2_ versus *τ*. The data for different *L* collapse and are fitted to Δ*S*_2_ = *s*_*c*_ln*τ* + const., yielding the non-equilibrium coefficient ${s}_{c}^{\,{\mbox{neq}}\,}=0.345(7)$. **c** Data collapse validating the universal scaling form in Eq. (2). Plotting Δ*S*_2_ − *s*_*c*_ ln *L* versus the scaling variable *τ*/*L* collapses all data onto a single master curve. The dashed line with the slope of 0.345 is plotted for comparison with the rescaled curve. The error bars represent the standard deviation.

Our method bypasses this limitation by exploiting the short-time relaxation dynamics from an AFM initial state, providing direct access to universal scaling with dramatically reduced resources (e.g., *L* = 9 and *τ* < 4). The results at *U*_*c*_ are summarized in Fig. 3. We observe the same sequence of scaling phenomena established in the non-interacting limit: Δ*S*_2_ shows a size-independent plateau governed by *τ* in the *τ* ≪ *L* regime as shown in Fig. 3a, and all data collapse when plotted against ln*τ* in Fig. 3b.

From the fit in Fig. 3b, we extract the non-equilibrium scaling coefficient: ${s}_{c}^{\,{\mbox{neq}}\,}=0.345(7)$. Crucially, in the non-equilibrium regime (1 ≲ *τ* ≪ *L*), the size dependence of Δ*S*_2_ is negligible for *L* ≥ 9. This enables the unambiguous extraction of the universal coefficient *s*_*c*_ using only modest system sizes and short imaginary times. Our high-precision result for *s*_*c*_ is significantly larger than the free-fermion prediction (*s*_*c*_ = 0.3116), a trend that aligns with recent findings in Ref. ^35^. This result highlights the stronger infrared entanglement signatures produced by the coupling between fermions and critical bosons, distinguishing the interacting GNY criticality from its free-fermion counterpart.

Finally, we validate the full scaling hypothesis of Eq. (2). As shown in Fig. 3c, plotting Δ*S*_2_ − *s*_*c*_ ln *L* versus *τ*/*L* results in the collapse of all data onto a single universal curve throughout the entire imaginary-time evolution regime *τ* ≳ 1. In addition, in the semi-log scale, the slope of the rescaled curve, which just corresponding to the scaling function ${{{\mathcal{F}}}}(\tau /{L}^{z})$, is close to 0.345, Fig. 3c, confirming that ${{{\mathcal{F}}}}(\tau /L)\to {s}_{c}{{{\rm{ln}}}}(\tau /L)$ as *τ* < *L*. These results validate the scaling ansatz and illustrate that the non-equilibrium relaxation timescale follows *τ*_neq_ ~ *L*, as expected for a QCP with dynamical exponent *z* = 1.

### Antiferromagnetic phase

In phases with spontaneous breaking of continuous symmetry, an additional logarithmic term related to the number of Goldstone modes (*N*_*G*_) appears^72^:

5 $${S}_{2}^{A}(L)=aL-({s}_{G}+{s}_{c}(\theta )) \; {{{\rm{ln}}}} \; L+\,{\mbox{const}}\,.+{{{\mathcal{O}}}}(1/L),$$

where *s*_*G*_ = − *N*_*G*_/2. The phase under study is characterized by the spontaneous breaking of *SU*(2) symmetry, resulting in *N*_*G*_ = 2 Goldstone modes (equivalent to two free real bosons). Notably, the *s*_*G*_ term arises from the bulk properties of the symmetry-broken phase regardless of the boundary shape, while *s*_*c*_ represents the specific corner contribution. By employing the SCEE subtraction scheme established above, we isolate *s*_*c*_ by canceling out the *s*_*G*_ term. For the antiferromagnetic (AFM) phase investigated here, each gapless boson is predicted to contribute approximately 0.0344 to the logarithmic coefficient for our specific geometry^21^. Consequently, this model serves as an ideal benchmark for extracting universal corner contributions via our non-equilibrium approach.

**Fig. 4:**
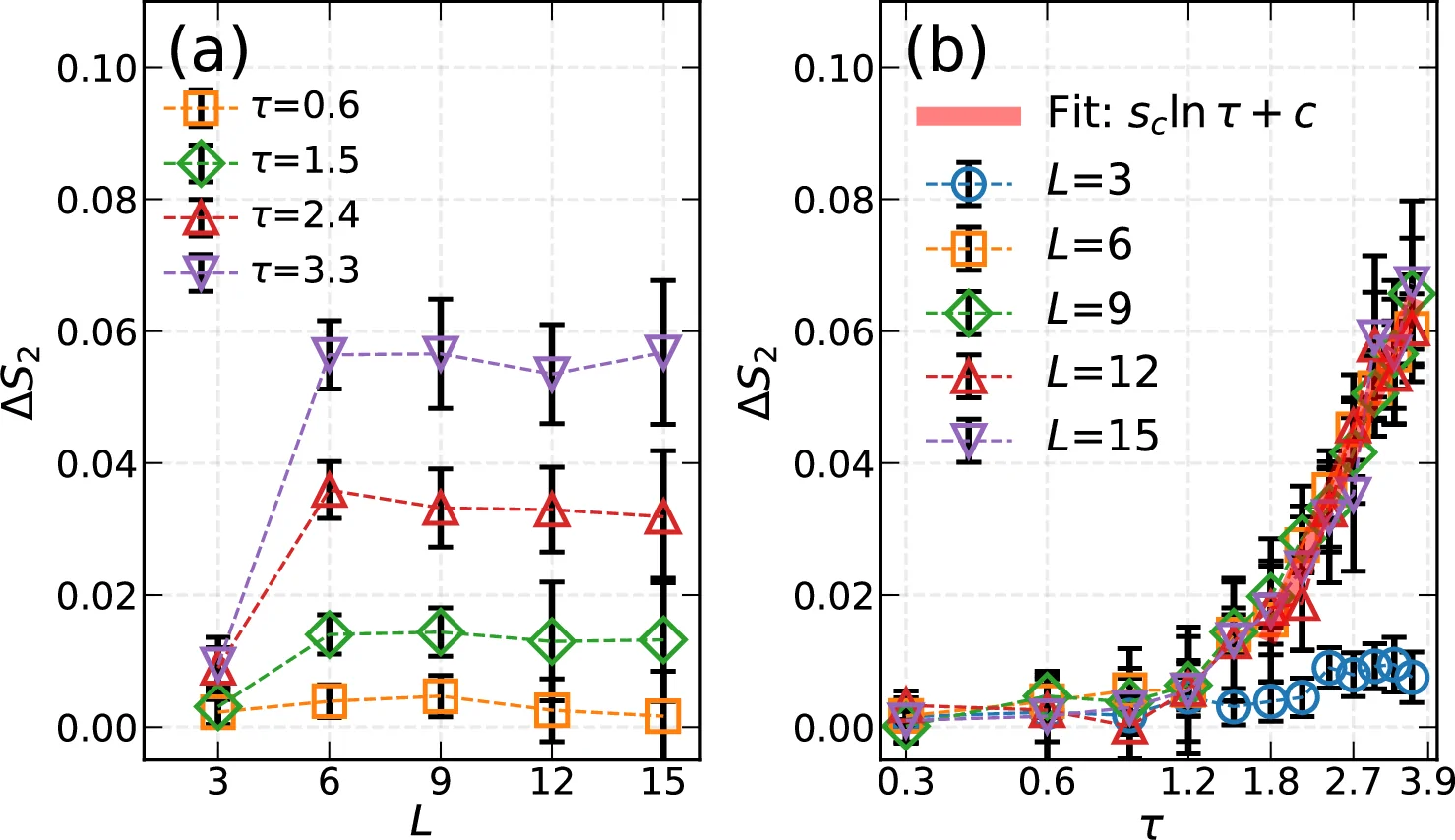
Non-equilibrium dynamics of Δ*S*_2_ at the AFM phase (*U* = 7). **a** Δ*S*_2_ versus *L* for different *τ*. In the non-equilibrium regime (*τ* ≪ *L*), Δ*S*_2_ exhibits a size-independent plateau. **b** Logarithm plotting of Δ*S*_2_ versus *τ*. The data for different *L* collapse and are fitted to Δ*S*_2_ = *s*_*c*_ln*τ* + const., yielding the non-equilibrium coefficient ${s}_{c}^{\,{\mbox{neq}}\,}=0.070(12)$.The error bars represent the standard deviation.

To investigate the dynamics deep within the AFM Mott insulator phase and avoid critical fluctuations associated with the GNY QCP, we perform simulations at large interaction strength (*U* = 7) starting from an AFM product state. The non-equilibrium dynamics exhibit scaling behavior qualitatively similar to the free fermion and QCP cases. As shown in Fig. 4a, in the non-equilibrium regime *τ* ≪ *L*, the system-size dependence of Δ*S*_2_ vanishes. In this regime, the corner contribution follows the scaling relation Δ*S*_2_ ~ *s*_*c*_ln*τ*. Fitting this form yields a universal coefficient *s*_*c*_ = 0.070(12), as shown in Fig. 4b. Results for *U* = 6 yield a consistent value of *s*_*c*_ = 0.069(10) (see Supplementary Fig. 3). These findings are in excellent agreement with the theoretical prediction for two Goldstone modes (2 × 0.0344 = 0.0688)^21^.

### Discussion and conclusion

We reveal a universal imaginary-time non-equilibrium scaling law for entanglement entropy in (2+1)D quantum critical systems. Through scaling analysis and numerical validation in free Dirac fermion systems, we establish that the corner entanglement contribution follows the general logarithmic relation Δ*S*_2_ = *s*_*c*_ln*τ* + const. within the non-equilibrium regime *τ*_0_ < *τ* ≪ *L*. Here, the coefficient *s*_*c*_ encodes the universal properties of the underlying CFT. Remarkably, a defining feature of this regime is that the entanglement growth is effectively independent of system size, providing a direct window into the extraction of critical data characteristic of the thermodynamic limit.

These non-equilibrium provides a powerful theoretical framework for extracting the universal entanglement properties that encode critical behavior at the QCP. Our protocol offers two decisive advantages over traditional equilibrium calculations. First, it drastically reduces computational cost. In QMC simulations, complexity scales with imaginary time; notably, in the presence of the sign problem, this scaling becomes exponential^73^. Therefore, by extracting universal data from the short-time dynamics, our approach effectively mitigates this bottleneck, making it feasible to study interacting models typically plagued by severe sign problems. Second, because the leading term lacks size-dependent corrections, our approach allows for the extraction of universal coefficients using smaller system sizes compared to traditional equilibrium methods. Applying this technique, we extract the universal corner contribution coefficient of the entanglement entropy at the GNY QCP in the honeycomb Hubbard model—a value for which no analytical solution currently exists. Our high-precision results reveal that the corner contribution at the GNY QCP is significantly enhanced relative to free fermions, highlighting the entanglement signatures of interacting fermions coupled to gapless bosons. This work opens a new avenue for efficiently calculating universal entanglement properties in exotic strongly correlated quantum systems, such as characterizing deconfined quantum critical points^74–79^ and Dirac spin liquids featuring emergent gauge fields^80–82^.

Besides the significance in theoretical and numerical aspects, the non-equilibrium scaling form would provide a theoretical framework for analyzing entanglement on quantum simulators and suggest immediate experimental applications. This progress is particularly timely; given the recent breakthroughs in realizing imaginary-time dynamics on superconducting quantum circuits, our proposed protocol can be directly implemented and verified on existing hardware^38–41^.

## Methods

We perform our calculations within the general framework of projector quantum Monte Carlo (PQMC) simulations^22,83^. To achieve high numerical stability and precision, we employ an incremental algorithm for entanglement entropy^35,71,84^. We isolate the universal logarithmic term using the Subtracted Corner Entanglement Entropy (SCEE) method^85–87^, which calculates the difference $\Delta {S}_{2}={S}_{2}^{B}-{S}_{2}^{A}$ between two regions with identical boundary lengths but distinct geometries. Specifically, region *B* has a smooth boundary (${s}_{c}^{B}=0$), while region *A* contains four sharp corners—two with *θ* = *π*/3 and two with *θ* = 2*π*/3 (see Fig. 1a). This subtraction directly isolates the corner contribution, where the universal coefficient *s*_*c*_ in Eq. (1) is given by ${s}_{c}={N}_{{{{\rm{f}}}}/{{{\rm{b}}}}}\times 2[{a}_{2}^{{{{\rm{f}}}}/{{{\rm{b}}}}}(\frac{\pi }{3})+{a}_{2}^{{{{\rm{f}}}}/{{{\rm{b}}}}}(\frac{2\pi }{3})]$, where *N*_f_ and *N*_b_ denote the number of fermion and boson species, respectively. Here, ${a}_{2}^{{{{\rm{f}}}}/{{{\rm{b}}}}}(\theta )$ represents the universal angle-dependent coefficients determined solely by the underlying CFT^21^. Further technical details are provided in the Supplementary Material (SM). See Supplementary Materials for more details of non-equilibrium PQMC simulation and more details of the algorithm and the numerical simulations in this study.

## Supplementary information


Supplementary Information
Transparent Peer Review file


## Data Availability

The data supporting the findings of this study have been deposited in Figshare at https://doi.org/10.6084/m9.figshare.31064440.

## Code availability

All numerical codes in this paper are available upon request to the corresponding authors (Z.X.L. and S.Y.).
