# Supplementary material for: Universal entanglement growth along imaginary time in quantum critical systems
Source: Nat Commun. 2026 Aug 3;17:9339. doi: 10.1038/s41467-026-75645-x (PMC13530133; doi:10.1038/s41467-026-75645-x)
Supplement: Supplementary file 1 [file 41467_2026_75645_MOESM1_ESM.pdf]

Supplemental Material for "Universal Entanglement Growth along Imaginary Time in Quantum Critical Systems"

Chang-Yu Shen[1,2], Shuai Yin[3,4],* and Zi-Xiang Li[1,2]†

[1] *Beijing National Laboratory for Condensed Matter Physics & Institute of Physics, Chinese Academy of Sciences, Beijing 100190, China*

[2] *University of Chinese Academy of Sciences, Beijing 100049, China*

[3] *Guangdong Provincial Key Laboratory of Magnetoelectric Physics and Devices, School of Physics, Sun Yat-sen University, Guangzhou 510275, China and*

[4] *School of Physics, Sun Yat-sen University, Guangzhou 510275, China*

I. PROJECTED QUANTUM MONTE CARLO AND RÉNYI ENTROPY

The Projected Quantum Monte Carlo (PQMC) method extracts the ground state $|\Psi_g\rangle$ from a trial wave function $|\Psi_T\rangle$ via imaginary-time evolution. Given a Hamiltonian $H = H_0 + H_U$, the projection is defined as:

$$|\Psi_g\rangle = \lim_{\tau\to\infty} e^{-\tau H} |\Psi_T\rangle . \tag{1}$$

To evaluate the imaginary-time propagator, we discretize the total projection time 2τ into N_τ slices of width $\Delta_\tau = 2\tau/N_\tau$. Since the non-interacting (H_0) and interacting (H_U) terms do not commute, we employ a first-order Trotter-Suzuki decomposition to approximate the partition function:

$$\mathcal{Z} = \langle\Psi_T| e^{-2\tau H} |\Psi_T\rangle = \langle\Psi_T| \left(e^{-\Delta_\tau H_0} e^{-\Delta_\tau H_U}\right)^{N_\tau} |\Psi_T\rangle + \mathcal{O}(\Delta_\tau^2). \tag{2}$$

Because H_U contains quartic fermionic operators, we decouple the interaction using a discrete $SU(2)$ Hubbard-Stratonovich (HS) transformation. By coupling the auxiliary fields s_i to the local charge density, the interacting propagator is expressed as:

$$e^{-\Delta_\tau \frac{U}{2}(n_{i,\uparrow}+n_{i,\downarrow}-1)^2} = \frac{1}{4}\sum_{s_i\in\{\pm1,\pm2\}} \gamma(s_i) e^{i\alpha\eta(s_i)(n_{i,\uparrow}+n_{i,\downarrow}-1)} + \mathcal{O}(\Delta_\tau^4), \tag{3}$$

where $\alpha = \sqrt{\Delta_\tau U/2}$ and the constants are given by $\gamma(\pm1) = 1+\sqrt{6}/3$, $\gamma(\pm2) = 1-\sqrt{6}/3$, $\eta(\pm1) = \pm\sqrt{2(3-\sqrt{6})}$, and $\eta(\pm2) = \pm\sqrt{2(3+\sqrt{6})}$.

Employing the Trotter-Suzuki decomposition and the discrete four-point Hubbard-Stratonovich (HS) transformation, the projection operator is expressed as a summation over auxiliary field configurations $\mathbf{s}$. This formulation enables the Monte Carlo sampling of the partition function $\mathcal{Z} = \langle\Psi_T|e^{-2\tau H}|\Psi_T\rangle$ [1]. To control discretization errors, we set the imaginary time step to $\Delta\tau = 0.1$ for interaction strengths $U = 3.8$ and $\Delta\tau = 0.03$ for $U = 6, 7$. The partition function is given by:

$$\mathcal{Z} = \sum_{\mathbf{s}} \mathcal{W}_{\mathbf{s}}, \quad \mathcal{W}_{\mathbf{s}} = \det\{\mathbf{P}^\dagger \mathbf{B}_{\mathbf{s}}(2\tau,0)\mathbf{P}\}, \tag{4}$$

where $\mathbf{P}$ is the matrix representation of the trial wavefunction $|\Psi_T\rangle$, and $\mathbf{B}_{\mathbf{s}}$ encodes the propagation of the kinetic term H_0 coupled with the auxiliary-field potential $V(\mathbf{s}_l)$.

A cornerstone of the PQMC framework is the equal-time Green's function at a specific imaginary time layer Θ:

$$\mathbf{G}_{\mathbf{s}}(\Theta) = \mathbb{I} - \mathbf{B}_{\mathbf{s}}(\tau,0)\mathbf{P}\left[\mathbf{P}^\dagger\mathbf{B}_{\mathbf{s}}(2\tau,0)\mathbf{P}\right]^{-1}\mathbf{P}^\dagger\mathbf{B}_{\mathbf{s}}(2\tau,\Theta). \tag{5}$$

The corresponding time-displaced Green's function $\mathbf{G}_{\mathbf{s}}(\tau_1,\tau_2)$ is defined as:

$$\mathbf{G}_{\mathbf{s}}(\tau_1,\tau_2) = \begin{cases} -[\mathbb{I}-\mathbf{G}_{\mathbf{s}}(\tau_1)]\mathbf{B}_{\mathbf{s}}^{-1}(\tau_2,\tau_1), & \tau_1<\tau_2, \\ \mathbf{B}_{\mathbf{s}}(\tau_1,\tau_2)\mathbf{G}_{\mathbf{s}}(\tau_2), & \tau_1>\tau_2. \end{cases} \tag{6}$$

The second Rényi entanglement entropy, S_2^A, is evaluated using the identity $e^{-S_2^A} = \mathrm{Tr}[\rho_A^2] = Z_2/Z_1^2 = \langle\det\mathbf{g}^A\rangle$. The Grover matrix $\mathbf{g}^A$, restricted to subsystem A, is constructed from the Green's functions of two independent replicas, $\mathbf{s}_1$ and $\mathbf{s}_2$, at the imaginary-time midpoint τ [2]:

$$\mathbf{g}^A_{\mathbf{s}_1,\mathbf{s}_2} = \mathbf{G}^A_{\mathbf{s}_1}(\tau)\mathbf{G}^A_{\mathbf{s}_2}(\tau) + [\mathbb{I}-\mathbf{G}^A_{\mathbf{s}_1}(\tau)][\mathbb{I}-\mathbf{G}^A_{\mathbf{s}_2}(\tau)]. \tag{7}$$

In the Monte Carlo sampling, the acceptance ratio for a single auxiliary-field update is given by

$$r = \prod_{\sigma} \left[\mathbb{I} + \Delta_x^\sigma (\mathbb{I} - \mathbf{G}_\mathbf{s}^\sigma(\Theta)) \right], \tag{8}$$

where Δ_x^σ denotes the rank-1 update matrix. To maintain computational efficiency, the imaginary-time-displaced Green's functions are updated following each accepted configuration change according to [3]:

$$\begin{cases} \mathbf{G}_{\mathbf{s}_1'}(\Theta) = \mathbf{G}_{\mathbf{s}_1}(\Theta) - \mathbf{G}_{\mathbf{s}_1}(\Theta)\mathbf{r}^{-1}\Delta[\mathbb{I} - \mathbf{G}_{\mathbf{s}_1}(\Theta)], \\ \mathbf{G}_{\mathbf{s}_1'}(\tau) = \mathbf{G}_{\mathbf{s}_1}(\tau) + \mathbf{G}_{\mathbf{s}_1}(\tau,\Theta)\mathbf{r}^{-1}\Delta\mathbf{G}_{\mathbf{s}_1}(\Theta,\tau), \\ \mathbf{G}_{\mathbf{s}_1'}(\Theta,\tau) = \mathbf{G}_{\mathbf{s}_1}(\Theta,\tau) + \mathbf{G}_{\mathbf{s}_1}(\Theta)\mathbf{r}^{-1}\Delta\mathbf{G}_{\mathbf{s}_1}(\Theta,\tau), \\ \mathbf{G}_{\mathbf{s}_1'}(\tau,\Theta) = \mathbf{G}_{\mathbf{s}_1}(\tau,\Theta) - \mathbf{G}_{\mathbf{s}_1}(\tau,\Theta)\mathbf{r}^{-1}\Delta[\mathbb{I} - \mathbf{G}_{\mathbf{s}_1}(\Theta)]. \end{cases} \tag{9}$$

Similarly, the Grover matrix $\mathbf{g}^A_{\mathbf{s}_1,\mathbf{s}_2}$ can be updated efficiently. An update in the configuration of the first replica, $\mathbf{s}_1 \to \mathbf{s}_1'$, results in the relation $\mathbf{g}^A_{\mathbf{s}_1',\mathbf{s}_2}(\mathbf{g}^A_{\mathbf{s}_1,\mathbf{s}_2})^{-1} = \mathbb{I} + \hat{a}\hat{b}$, with:

$$\hat{a} = \mathbf{G}_{\mathbf{s}_1}(\tau,\Theta)\mathbf{r}^{-1}\Delta, \quad \hat{b} = \mathbf{G}_{\mathbf{s}_1}(\Theta,\tau)[2\mathbf{G}_{\mathbf{s}_2}(\tau) - \mathbb{I}](\mathbf{g}^A_{\mathbf{s}_1,\mathbf{s}_2})^{-1}. \tag{10}$$

An analogous expression holds for updates to the second replica, $\mathbf{s}_2 \to \mathbf{s}_2'$, where:

$$\hat{a} = [2\mathbf{G}_{\mathbf{s}_1}(\tau) - \mathbb{I}]\mathbf{G}_{\mathbf{s}_2}(\tau,\Theta), \quad \hat{b} = \mathbf{r}^{-1}\Delta\mathbf{G}_{\mathbf{s}_2}(\Theta,\tau)(\mathbf{g}^A_{\mathbf{s}_1,\mathbf{s}_2})^{-1}. \tag{11}$$

By exploiting the rank-1 structure of Δ, only a single row or column slice of the matrices $\hat{a}$ and $\hat{b}$ is required to enable efficient and rapid updates to the Grover matrix $\mathbf{g}^A$.

II. INCREMENTAL ALGORITHM FOR ENTANGLEMENT ENTROPY

The Rényi entropy S_2 exhibits extensive scaling with subsystem size. Although Grover's method within the DQMC framework provides an unbiased estimator for e^{-S_2} [2], the exponential decay of this quantity with system size L results in a severe signal-to-noise ratio problem. To mitigate the exponentially growing relative statistical errors, we employ the incremental algorithm. We decompose the expectation value $O \equiv \det \mathbf{g}^A$ into a product of incremental ratios along a monotonically increasing path $\boldsymbol{\lambda} = \{\lambda_0, \lambda_1, \ldots, \lambda_N\}$ with $\lambda_0 = 0$ and $\lambda_N = 1$ [3, 4]:

$$\langle O \rangle = \prod_{n=0}^{N-1} \frac{\sum_i P_i O_i^{\lambda_n} O_i^{\Delta_\lambda}}{\sum_i P_i O_i^{\lambda_n}}. \tag{12}$$

For systematic analysis, we utilize a linear parameterization $\lambda_n = n/N_\lambda$, where $\Delta_{\lambda_n} = 1/N_\lambda$ is the incremental step size. As the lattice size L increases, the number of intermediate λ-points must be increased to constrain the statistical error. As illustrated in Supplementary Figure 1(a), the logarithm of the determinant, $\ln(\det \mathbf{g}^A)$, follows a normal distribution $\mathcal{N}(\mu, \sigma^2)$. By fitting the data (Supplementary Figure 1(b)), we extract the scaling behavior of the mean $\mu(L) \propto L^\alpha$ and standard deviation $\sigma(L) \propto L^\beta$.

Crucially, the coefficient of variation for the raw determinant, $CV[\det \mathbf{g}^A] = \sqrt{e^{\sigma^2} - 1}$, grows exponentially with L. In contrast, the coefficient of variation for the incremental step scales as $CV[(\det \mathbf{g}^A)^{1/N_\lambda}] = \sqrt{e^{\sigma^2/N_\lambda^2} - 1}$. Consequently, to ensure numerical precision, we select $N_\lambda(L) \approx 0.2L^{1.7} > \sigma(L)$. Specifically, we use $N_\lambda = \{2, 5, 9, 15, 21, 28\}$ for system sizes $L = \{3, 6, 9, 12, 15, 18\}$, respectively.

At each step n, the effective weight and observable are defined as:

$$\begin{cases} \mathbf{P}_i^{(n)} \equiv P_i O_i^{\lambda_n}, \\ O_i^{(n)} \equiv O_i^{\Delta_{\lambda_n}}. \end{cases} \tag{13}$$

The modified acceptance ratio for a single auxiliary field flip is $R_{\lambda_n} = r \cdot (\det \Gamma_A)^{\lambda_n}$, where r is the standard PQMC update ratio and $\Gamma_A \equiv \mathbb{I} + \hat{b}\hat{a}$ accounts for the subsystem contribution.

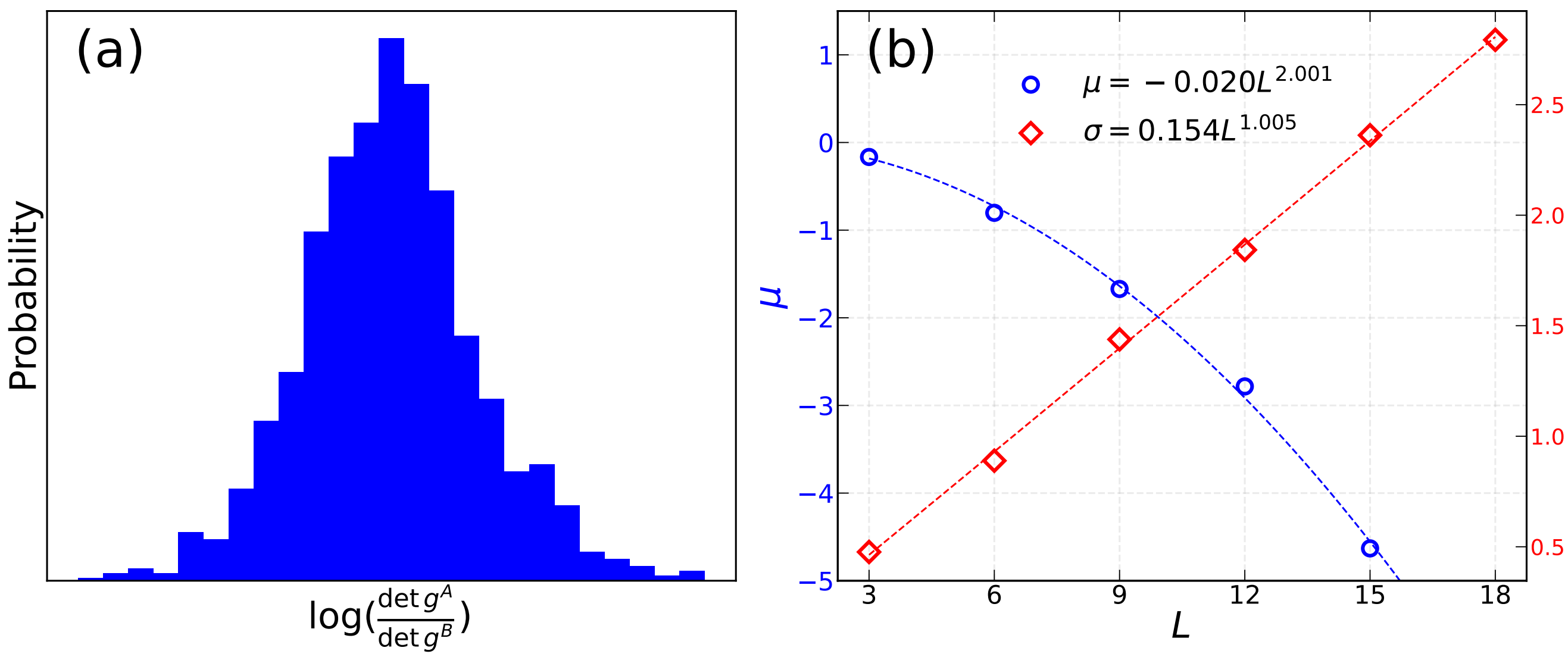

Supplementary Figure 1. (a) Sampling distribution of $\log(\det \mathbf{g}^A/\det \mathbf{g}^B)$, which follows a normal distribution $\mathcal{N}(\mu, \sigma^2)$. (b) Scaling of the statistical mean μ (left) and standard deviation σ (right) with system size L. The solid lines represent fits to the scaling relations $\mu \approx -0.02L^{1.995}$ and $\sigma \approx 0.165L^{0.981}$.

III. SUBTRACTED CORNER ENTANGLEMENT ENTROPY

The universal corner contribution to the second Rényi entropy is isolated through the subtracted corner entanglement entropy $\Delta S_2 = S_2^A - S_2^B$. Here, S_2^A and S_2^B denote the entanglement entropies for subsystems with geometries A and B, respectively, which are carefully constructed to share identical boundary lengths but differ only in the presence of corners. This subtraction scheme effectively cancels the non-universal area-law contributions, leaving only the universal corner term. To circumvent numerical instabilities and the prohibitive computational overhead inherent in independent estimations of S_2^A and S_2^B, we directly sample the exponential difference $e^{-\Delta S_2}$ through a ratio of determinants [5–7]:

$$e^{-(S_A - S_B)} = \frac{\sum_{\mathbf{s}_1,\mathbf{s}_2} P_{\mathbf{s}_1,\mathbf{s}_2} \det \mathbf{g}^B_{\mathbf{s}_1,\mathbf{s}_2} \cdot \frac{\det \mathbf{g}^A_{\mathbf{s}_1,\mathbf{s}_2}}{\det \mathbf{g}^B_{\mathbf{s}_1,\mathbf{s}_2}}}{\sum_{\mathbf{s}_1,\mathbf{s}_2} P_{\mathbf{s}_1,\mathbf{s}_2} \det \mathbf{g}^B_{\mathbf{s}_1,\mathbf{s}_2}}, \tag{14}$$

where $\mathbf{g}^{A/B}$ are the Grover matrices restricted to subsystems A and B, constructed from the mid-time Green's functions of two independent auxiliary field configurations $\mathbf{s}_1, \mathbf{s}_2$. In the combined Incremental and Subtracted (ICR-SCEE) method, we use the linear ramp of incremental path $\lambda_n \in [0, 1]$ to ensure the accuracy as:

$$\begin{cases} \mathcal{P}_i^{(n)} \equiv P_{\mathbf{s}_1,\mathbf{s}_2} (\det \mathbf{g}^B_{\mathbf{s}_1,\mathbf{s}_2})^{1-\lambda_n} (\det \mathbf{g}^A_{\mathbf{s}_1,\mathbf{s}_2})^{\lambda_n}, \\ \mathcal{O}_i^{(n)} \equiv \left(\dfrac{\det \mathbf{g}^A_{\mathbf{s}_1,\mathbf{s}_2}}{\det \mathbf{g}^B_{\mathbf{s}_1,\mathbf{s}_2}} \right)^{\Delta_{\lambda_n}}. \end{cases} \tag{15}$$

This formulation ensures a smooth interpolation between the geometries of subsystems A and B. The corresponding ICR-SCEE update ratio for an auxiliary field flip is:

$$R_{\lambda_n} = r \cdot \det(\Gamma_A)^{\lambda_n} \cdot \det(\Gamma_B)^{1-\lambda_n}, \tag{16}$$

where r is the standard PQMC update ratio, and $\Gamma_{A/B}$ represent the subsystem-dependent update factors for $\det \mathbf{g}^{A/B}$ derived from rank-1 matrix modifications.

IV. BENCHMARK TO THE RESULTS OF FREE DIRAC FERMIONS AND GAPLESS BOSONS

To provide a rigorous validation of our numerical results, we benchmark the corner contribution to the second Rényi entropy, s_c, against established values for free relativistic theories. In the low-energy limit, the Dirac semi-metal and

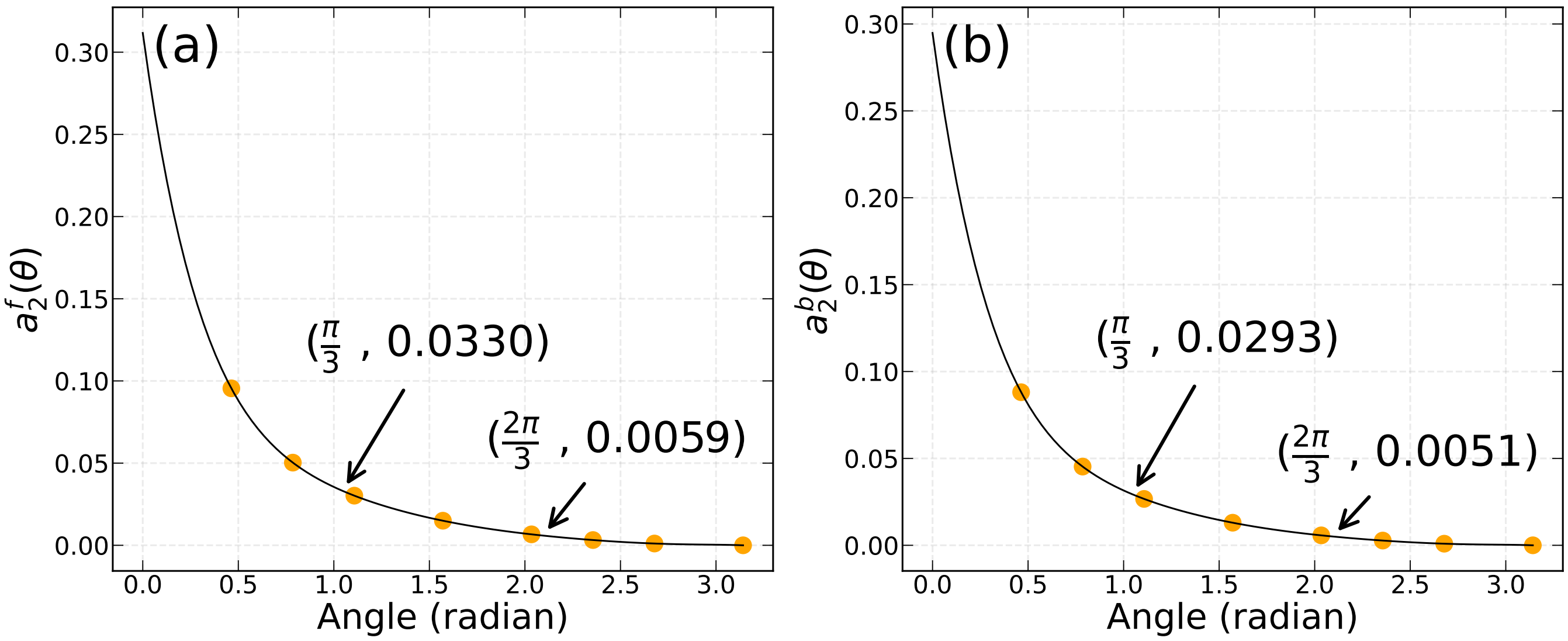

Supplementary Figure 2. Corner contributions to the second Rényi entropy ($\alpha = 2$) for (a) Dirac fermions and (b) free bosons. The numerical data points correspond to the high-precision results reported in Ref. [8]. To obtain the corner function values at $\theta = \pi/3$ and $2\pi/3$ required for our analysis, we perform a controlled polynomial interpolation of the tabulated data. This method preserves the smooth angular dependence observed in the corner function, enabling precise evaluation of $a_2^{\mathrm{f/b}}(\pi/3)$ and $a_2^{\mathrm{f/b}}(2\pi/3)$.

the antiferromagnetic (AFM) phases of our model are described by free Dirac fermions and gapless relativistic bosons, respectively. Given that the angles accessible in our calculations generally do not align with the discrete set of angles tabulated in Ref. [8], we perform a controlled polynomial interpolation of the published numerical data, as illustrated in Supplementary Figure 2. This interpolation method ensures continuity with the smooth-angle dependence observed in the corner function, enabling precise evaluation of $a_2^{\mathrm{f/b}}(\pi/3)$ and $a_2^{\mathrm{f/b}}(2\pi/3)$. Here, $a_2^{\mathrm{f/b}}(\theta)$ denotes the universal coefficient of the logarithmic corner correction to the Rényi entropy S_2 for a single flavor of Dirac fermions (f) or gapless relativistic complex bosons (b), respectively.

The honeycomb lattice hosts four flavors of degenerate gapless Dirac fermions, accounting for both spin and valley degrees of freedom. In the Dirac semi-metal phase, the total corner contribution s_c is obtained by summing the contributions from all flavors across the relevant opening angles:

$$s_c = 4 \times 2 \left[a_2^{\mathrm{f}}(\pi/3) + a_2^{\mathrm{f}}(2\pi/3)\right] = 0.3116. \tag{17}$$

Here, the factor of 4 accounts for the four fermion flavors, while the factor of 2 represents the two pairs of corners (with angles $\pi/3$ and $2\pi/3$) present in our entanglement geometry.

In the antiferromagnetic (AFM) phase, the low-energy physics is governed by two Goldstone modes corresponding to two real free bosons (equivalent to one complex free boson). The associated corner contributions are given by

$$\frac{1}{2} \times 2 \left[a_2^{\mathrm{b}}(\pi/3) + a_2^{\mathrm{b}}(2\pi/3)\right] = 0.0344. \tag{18}$$

The prefactor $\frac{1}{2}$ accounts for the equivalence between two real bosons and one complex boson, while the summation captures the angular dependence of the corner function for each Goldstone mode.

V. ADDITIONAL RESULT OF QUANTUM MONTE-CARLO SIMULATION

To corroborate the universality of our non-equilibrium protocol within the symmetry-broken phase, we extended our projector quantum Monte Carlo (PQMC) simulations to an interaction strength of $U = 6$. This parameter regime lies deep within the antiferromagnetic (AFM) Mott insulating phase but possesses distinct microscopic energy scales compared to the $U = 7$ case presented in the main text. In the AFM phase, the continuous $SU(2)$ spin rotational symmetry is spontaneously broken down to $U(1)$, resulting in $N_G = 2$ gapless Goldstone modes (spin

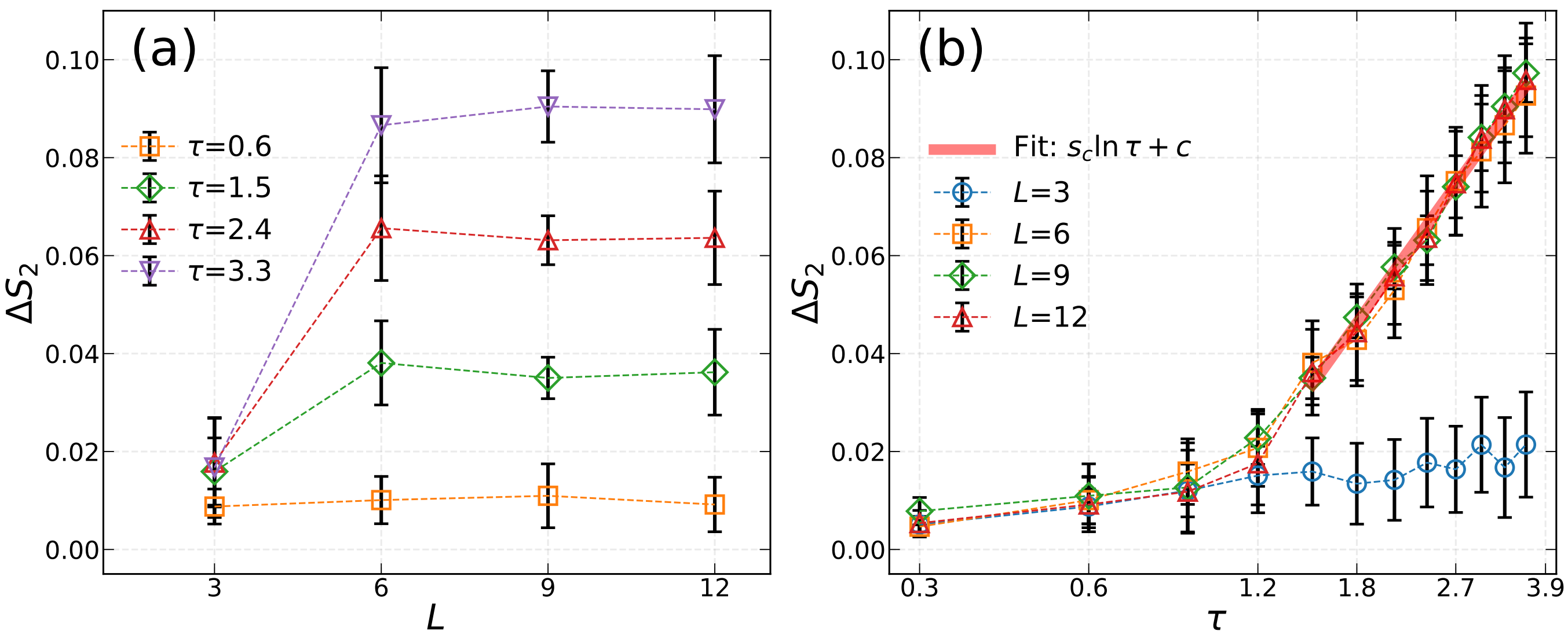

Supplementary Figure 3. Non-equilibrium dynamics of ΔS_2 at $U = 6$. (a) ΔS_2 vs. system size L for various τ, showing size-independent plateau. (b) Logarithmic plot of ΔS_2 vs. τ, revealing linear scaling in non-equilibrium regime. The extracted corner contribution is $s_c = 0.069(10)$.

waves). These modes are expected to dominate the universal corner entanglement contribution, theoretically predicted to be $s_c = 0.0688$, arising from the sum of two free bosonic modes (2×0.0344) [8].

As illustrated in Supplementary Figure 3(a), the subtracted corner entanglement entropy ΔS_2 exhibits the characteristic size-independent plateau in the short-time relaxation regime ($\tau \ll L$). This confirms that, the short-time dynamics are governed by local entanglement spreading rather than finite-size geometry. By performing a logarithmic fit to the scaling ansatz $\Delta S_2(\tau) \sim s_c \ln \tau$ over the temporal window corresponding to this plateau, as shown in Supplementary Figure 3(b), we extract a non-equilibrium universal coefficient of $s_c = 0.069(10)$.

These results are significant physical consequence for two primary reasons. First, the value obtained is consistent with $s_c = 0.070(12)$ measured at a stronger coupling ($U = 7$) within error bars. Second, it demonstrates excellent quantitative agreement with the rigorous field-theoretic prediction for free relativistic bosons. The stability of s_c across a range of Hubbard repulsions U serves as a robust validation of our methodology. It confirms that the imaginary-time dynamics effectively filter out non-universal microscopic details, providing a reliable and efficient probe for the universal entanglement features of phases characterized by the spontaneous breaking of continuous symmetries.

* yinsh6@mail.sysu.edu.cn
† zixiangli@iphy.ac.cn

[1] F. Assaad and H. Evertz, World-line and determinantal quantum Monte Carlo methods for spins, phonons and electrons, in *Computational Many-Particle Physics* (Springer Berlin Heidelberg, Berlin, Heidelberg, 2008) pp. 277–356.
[2] T. Grover, Phys. Rev. Lett. **111**, 130402 (2013).
[3] G. Pan, Y. Da Liao, W. Jiang, J. D'Emidio, Y. Qi, and Z. Y. Meng, Phys. Rev. B **108**, L081123 (2023).
[4] J. D'Emidio, R. Orús, N. Laflorencie, and F. de Juan, Phys. Rev. Lett. **132**, 076502 (2024).
[5] J. Helmes, *An entanglement perspective on phase transitions, conventional and topological order*, Ph.D. thesis, Universität zu Köln (2017).
[6] Z. H. Liu, Y. Da Liao, G. Pan, M. Song, J. Zhao, W. Jiang, C.-M. Jian, Y.-Z. You, F. F. Assaad, Z. Y. Meng, and C. Xu, Phys. Rev. Lett. **132**, 156503 (2024).
[7] Y. Da Liao, M. Song, J. Zhao, and Z. Y. Meng, Phys. Rev. B **110**, 235111 (2024).
[8] J. Helmes, L. E. Hayward Sierens, A. Chandran, W. Witczak-Krempa, and R. G. Melko, Phys. Rev. B **94**, 125142 (2016).
