# Supplementary material for: Universal entanglement growth along imaginary time in quantum critical systems
Source: Nat Commun. 2026 Aug 3;17:9339. doi: 10.1038/s41467-026-75645-x (PMC13530133; doi:10.1038/s41467-026-75645-x)
Supplement: Supplementary file 2 [file 41467_2026_75645_MOESM2_ESM.pdf]

nature portfolio

Peer Review File

Universal Entanglement Growth along Imaginary Time in Quantum Critical Systems

Corresponding Author: Professor Zi-Xiang Li

This file contains all reviewer reports in order by version, followed by all author rebuttals in order by version.

Version 1:

Reviewer comments:

Reviewer #1

(Remarks to the Author)
This manuscript reports a conceptually new and technically important advance at the interface of quantum information, non-equilibrium critical phenomena, and computational many-body physics. The authors identify a universal short-imaginary-time scaling law for the corner contribution to the second Renyi entropy in 2+1D critical systems. In my view, this is the central conceptual contribution of the paper: universal corner entanglement data, usually extracted only after expensive ground-state convergence, can instead be obtained from the early stages of imaginary-time relaxation.

The numerical evidence is strong and carefully structured. The free-fermion benchmark is particularly convincing: the non-equilibrium estimate $s_c^{\mathrm{neq}}=0.3111(15)$ agrees extremely well with the equilibrium extrapolation $s_c^{\mathrm{eq}}=0.3116(13)$ and with the known CFT expectation $s_c=0.3116$. This establishes that the proposed short-time protocol reproduces the correct universal coefficient before the system reaches full equilibrium. The paper then moves beyond benchmarking and applies the method to the interacting Gross-Neveu-Yukawa critical point of the honeycomb Hubbard model, where the authors extract $s_c^{\mathrm{neq}}=0.345(7)$, and further validate the framework in the antiferromagnetic phase, obtaining $s_c=0.070(12)$ at $U=7$ and $0.069(10)$ at $U=6$, both consistent with the expected Goldstone-mode value 0.0688. Taken together, these results make a compelling case that the protocol is both universal and practically useful.

A major strength of the manuscript is that the advance is not only conceptual but also methodological. The authors combine projector QMC with an incremental estimator and a subtracted-corner-entanglement construction to isolate the universal logarithmic term while controlling the severe variance problems that usually plague Renyi-entropy calculations. The supplementary material makes the numerical framework sufficiently transparent: it spells out the PQMC estimator, the rank-1 update structure for the Grover matrix, the incremental decomposition used to tame the exponential growth of fluctuations, and the scaling choice $N_\lambda(L)\approx 0.2L^{1.7}$ used in practice. This level of methodological detail strengthens confidence in the numerical claims and increases the likely impact of the work on future studies of entanglement in interacting quantum matter.

I also find the broader significance convincing. The paper is not merely another numerical estimate of a corner coefficient. Rather, it establishes a new route to universal entanglement spectroscopy in higher-dimensional critical systems, including regimes where equilibrium calculations are especially costly. The authors explicitly show that the method captures the interacting GNY critical point, where no analytical value is available for the reported geometry, and they motivate extensions to more exotic settings such as deconfined criticality, Dirac spin liquids, and platforms implementing imaginary-time evolution on quantum hardware. This breadth, combined with the solid many-body numerics and the clear universal message, makes the manuscript well suited for Nature Communications.

In summary, I recommend publication in Nature Communications. The work delivers a genuine conceptual advance, a numerically validated universal scaling law, and a broadly useful computational protocol for extracting entanglement data in interacting quantum critical systems. I expect it to be of substantial interest to specialists working in quantum many-body physics, entanglement theory, critical phenomena, and quantum simulation.

(Remarks on code availability)

Reviewer #2

(Remarks to the Author)
The paper entitled “Universal Entanglement Growth along Imaginary Time in Quantum Critical Systems” studies the second Rényi entropy in the ground states of two-dimensional critical systems. The authors focus on the Hubbard model on a honeycomb lattice at half filling. They consider imaginary-time evolution, which can be interpreted as a cooling process that drives the system toward its ground state at long imaginary times. The authors aim to determine the universal contribution to the entanglement entropy in equilibrium, namely the logarithmic correction arising from the sharp corners of the considered subsystem. For this reason, they propose a well-justified ansatz for the nonequilibrium scaling of this contribution with imaginary time and (linear) subsystem size. The authors argue that for short imaginary times, when correlations have not yet reached the boundary of the subsystem, the scaling should depend on the correlation length rather than the subsystem size. The authors validate this ansatz through a series of numerical calculations, considering the free-fermion, antiferromagnetic, and critical regimes.

I found the paper well written and the results interesting. The outcome of this study is promising, as the authors demonstrate that the universal contribution to the entanglement entropy in equilibrium can be extracted from relatively short imaginary times and small subsystem sizes in imaginary-time evolution. Therefore, I believe that the paper meets the high standards of the journal and can be published in its current form.

However, I do have a small comment. In the introduction, the authors use the term “equilibrium ground state”. Is this a conventional term in the context of imaginary-time evolution? I find it somewhat misleading. If this is not a commonly used term, I would suggest replacing it simply with “ground state” or “equilibrium low-temperature state”.

There are also several misspellings in the paper. For example, “product initial stage” should likely read “product initial state”, while “QFT” appears instead of “CFT”. Moreover, the abbreviation “EE”, which I believe stands for entanglement entropy, does not seem to be introduced before being used.

In the introduction, the authors also write “We report a fundamental discovery,” which appears somewhat overstated for a scientific manuscript.

(Remarks on code availability)

Open Access This Peer Review File is licensed under a Creative Commons Attribution 4.0 International License, which permits use, sharing, adaptation, distribution and reproduction in any medium or format, as long as you give appropriate credit to the original author(s) and the source, provide a link to the Creative Commons license, and indicate if changes were made.
In cases where reviewers are anonymous, credit should be given to 'Anonymous Referee' and the source.
The images or other third party material in this Peer Review File are included in the article’s Creative Commons license, unless indicated otherwise in a credit line to the material. If material is not included in the article’s Creative Commons

license and your intended use is not permitted by statutory regulation or exceeds the permitted use, you will need to obtain permission directly from the copyright holder.
To view a copy of this license, visit https://creativecommons.org/licenses/by/4.0/

Response to Reviewers

Response to Reviewer #1

Comment 1:

"This manuscript reports a conceptually new and technically important advance... I recommend publication in Nature Communications."

Response:

We are deeply grateful for your thorough reading and insightful summary of our work. We are pleased that you recognize the potential of our short-imaginary-time protocol to impact future studies of entanglement in interacting quantum matter. Your endorsement is greatly appreciated. We are grateful that you recommend publication of our manuscript in Nature Communications.

Response to Reviewer #2

Comment 1:

"However, I do have a small comment. In the introduction, the authors use the term 'equilibrium ground state'. Is this a conventional term in the context of imaginary-time evolution? ... I would suggest replacing it simply with 'ground state'..."

Response:

We agree with the reviewer that the term "equilibrium ground state" is potentially misleading and redundant in this context. Following your suggestion, we have replaced all instances of "equilibrium ground state" with simply **"ground state"** throughout the revised manuscript (including the abstract and introduction). This improves the clarity and precision of our terminology regarding imaginary-time evolution.

Comment 2:

"There are also several misspellings in the paper. For example, 'product initial stage' should likely read 'product initial state', while 'QFT' appears instead of 'CFT'. Moreover, the abbreviation 'EE', which I believe stands for entanglement entropy, does not seem to be introduced before being used."

Response:

We apologize for these oversights. We have corrected them as follows:

1. Changed **"product initial stage"** to **"product initial state"**.
2. Corrected the typo **"QFT"** to **"CFT"** (Conformal Field Theory).

3. We have now explicitly introduced the abbreviation **"EE"** (Entanglement Entropy) upon its first appearance in the introduction.

Comment 3:

"In the introduction, the authors also write 'We report a fundamental discovery,' which appears somewhat overstated for a scientific manuscript."

Response:

We appreciate this critique regarding scientific tone. While we believe the result is foundational, we agree that the phrasing was overly assertive. We have revised this sentence to be more measured. The revised text now reads: **"We identify a universal scaling law..."** (or similar phrasing depending on the exact flow of the paragraph), removing the subjective claim of a "discovery."
